# Evaluation of Multidrug Resistance of *Salmonella* Isolated from Pork Meat Obtained from Traditional Slaughter Systems in Romania

**DOI:** 10.3390/microorganisms12112196

**Published:** 2024-10-30

**Authors:** Alexandra Tăbăran, Sorin Danel Dan, Liora Mihaela Colobaţiu, Marian Mihaiu, Sergiu Condor, Rodica Mărgăoan, Oana Lucia Crişan-Reget

**Affiliations:** 1 Department of Animal Husbandry and Public Health, Faculty of Veterinary Medicine Cluj-Napoca, University of Agricultural Sciences and Veterinary Medicine Cluj, 400372 Cluj-Napoca, Romania; sorindan@usamvcluj.ro (S.D.D.); mihaiu.marian@usamvcluj.ro (M.M.); sergiu.condor@student.usamvcluj.ro (S.C.); rodica.margaoan@usamvcluj.ro (R.M.); oana.reget@usamvcluj.ro (O.L.C.-R.); 2 Department of Medical Devices, Faculty of Pharmacy, Iuliu Hatieganu University of Medicine and Pharmacy, Victor Babes Street No. 8, 400012 Cluj-Napoca, Romania; mihaiu.mihaela@umfcluj.ro

**Keywords:** antimicrobial resistance (AMR), *Salmonella*, traditional slaughtering, food safety

## Abstract

Antibiotic resistance among pathogenic bacteria in humans and animals poses a critical public health challenge, leading to diminished effectiveness of existing antimicrobial treatments. Notably, animal-derived food products are significant vectors for the transmission of resistant bacteria to humans, with *Salmonella* species being predominant culprits in foodborne illnesses. This study investigates the prevalence and antibiotic resistance patterns of *Salmonella* serovars isolated from traditionally sourced pork meat in Romania. Over a four-year period, 208 pork samples were collected using standardized protocols during traditional slaughtering practices. *Salmonella* spp. were isolated following ISO 6579:2002 guidelines and confirmed using biochemical assays and PCR. Serotyping was performed using specific antisera, and antimicrobial susceptibility testing was conducted through the standard disk diffusion method, assessing 11 antibiotics. Results indicated a 23.07% prevalence of Salmonella, with 48 isolates categorized into eight serovars, primarily *S*. Infantis (*n* = 15), *S*. Typhimurium (*n* = 15), and *S*. Derby (*n* = 11). PCR results confirmed the presence of *Salmonella* by detecting the *hilA* and *ompC* genes, with 31.25% of isolates being positive for the *Typhimurium*-specific sequence. Notably, 93.75% of the isolates were multidrug-resistant (MDR), exhibiting high resistance rates against streptomycin (91.66%) (>10 µg), tetracycline (83.33%) (>30 µg), and sulfamethoxazole (68.75%) (>300 µg). More than 60% of MDR isolates displayed resistance to five or more antibiotics. These findings underscore the need for coordinated control measures in the pork production chain to combat the spread of *Salmonella* and protect public health. Enhanced surveillance and intervention strategies are crucial for addressing antibiotic resistance and reducing the risk of foodborne illnesses linked to contaminated animal products.

## 1. Introduction

Antibiotic resistance among pathogenic agents, both in animals and humans, has become a matter of significant concern, leading to a limited duration of effectiveness of antimicrobial compounds [1]. Furthermore, the development of new and effective antimicrobial compounds is unlikely to occur at a sufficient rate. Consequently, we face the inability to adopt new methods to combat bacterial pathogens—or to develop new ways to delay the acquisition of resistance. This has been and will remain a significant issue in therapeutics [2]. Certain authors have forecasted that by the year 2050, antimicrobial-resistant pathogens will be accountable for causing 10 million fatalities globally [3]. Human contamination with these resistant bacteria represents a public health concern. There are numerous ways in which these bacteria can contaminate humans, some of which occur through food, particularly animal-derived foods, causing illness directly or acting as a potential source of antibiotic resistance for human pathogens [4].

Antibiotic resistance associated with the food chain is currently a major concern for many stakeholders in the production chain, especially the traditional ones which lack efficient control measures. Numerous studies have demonstrated the significant role that animal-derived products play in the occurrence of foodborne illnesses and various microbes resistant to antimicrobial products [5,6]. *Salmonella* species are the most important microbial agents responsible for foodborne illnesses, with salmonellosis still being the most widespread type of foodborne illness affecting humans, with clinical manifestations ranging from asymptomatic stages to severe issues [1,7,8]. Consuming contaminated food, such as chicken, beef, pork, eggs, cheese, seafood, fruits, beverages, or vegetables, is linked to the majority of bacterial infections [9,10,11]. However, most infections caused by microbes resistant to multiple antimicrobial agents are due to the ingestion of contaminated animal-derived foods [12].

*Salmonella* species are commonly encountered in the gastrointestinal tract of animals and are widely distributed in the environment, particularly among livestock such as chickens, pigs, and cattle [13]. This genus comprises two main species: *S. enterica* and *S. bongori*. In swine, clinical salmonellosis has been predominantly associated with two serovars: *S.* Choleraesuis, especially the Kunzendorf variety, and *S.* Typhimurium [14]. The predominant causes of the clinical disease are the *S.* Choleraesuis serovar, which causes systemic disease with septicemia (frequently accompanied by pneumonia); and *S.* Typhimurium and *S.* Enteritidis, which produce enteric disease, which is characterized by diarrhea [15,16]. Thus, *S.* Enteritidis and *S.* Typhimurium are recognized as the predominant serotypes responsible for foodborne outbreaks [16].

Over time, shifts and developments within the pork industry may have led to new challenges and barriers in controlling *Salmonella* along the food chain [15,16]. Previous research underscores the need for synchronized control strategies and standardized monitoring programs to manage *Salmonella* effectively in swine [17]. Such measures are essential for preventing clinical disease outbreaks in pigs and for protecting consumers by controlling subclinical *Salmonella* carriage and shedding. As of January 2020, global pig populations were estimated at around 677.6 million [18]. Over the past century, the pig production industry has shifted significantly, moving from small herds to large-scale facilities that accommodate vast numbers of animals [19,20]. The rapid expansion of intensive farming practices may have contributed to emerging challenges in the management and control of swine salmonellosis [21,22]. Additionally, because many infected pigs are asymptomatic carriers, data on the incidence of *Salmonella* infections in pigs are scarce and frequently just scratch the surface [23].

The prevalence of Salmonella and associated risk factors within the pig value chain have been extensively studied in many areas throughout the United States and across the European Union. It is now evident that infections at the farm level, in pigs intended for slaughter, are the source of the *Salmonella* contamination of pig carcasses [24]. Additionally, cross-contamination during slaughtering has been shown to play a significant role in the presence of *Salmonella* in carcasses [25]. Both health and financial repercussions result from this contamination: In the Netherlands and Germany, ingestion of infected pork or pork products is thought to be the cause of 15–20% of human cases of *Salmonella* infections [25,26]. A study in Ireland [24] revealed significant Salmonella contamination in pig populations, highlighting both the ongoing challenges of prevalence on farms and the improvements in hygiene practices at abattoirs. The article specifically addresses the challenges of reducing Salmonella in carcass samples and highlights the issue of antimicrobial resistance, noting that over 40% of the strains were multidrug-resistant. The traditional rearing practices, especially the conventional slaughtering methods employed for pork and chicken, still practiced in Romania, albeit primarily for private consumption, may pose an elevated risk of contamination. However, there is a lack of available data regarding antimicrobial-resistant strains’ potential prevalence in meat. Research endeavors are imperative, particularly considering recent reports indicating the presence of *Salmonella* strains in poultry meat exhibiting resistance to extended-spectrum cephalosporins [27]. These bacteria have been associated with treatment failures in human cases, necessitating the utilization of alternative antibiotics to manage infections [28,29]. The effectiveness of therapies for infections in humans may be compromised by *Salmonella* strains developing antibiotic resistance and the appearance of virulent clones, posing significant challenges in disease control and representing a grave threat to public health worldwide [30]. Consequently, the World Health Organization has classified *Salmonella* as a “priority pathogen” and aims to direct and support scientific study and development efforts targeted at finding new antibiotics to treat it [31,32].

Given this context, the present study aimed to evaluate the frequency, patterns of resistance to antibiotics, and screening for resistance genes within *Salmonella* serovars isolated from traditionally sourced pork meat collected from various traditional processing facilities located in the northwestern region of Romania.

## 2. Materials and Methods

### 2.1. Collection of the Sample

The experiment involved 208 pork meat samples collected between 2019 and 2023 in the northwest region of Transylvania (Cluj and Alba county), primarily during the winter months when traditional slaughtering practices are prevalent in Romania. Specimens were collected following a standardized protocol, using the destructive method (Commission Regulation (EC) no. 1441/2007) [33]. Briefly, four tissue samples representing a total area of 20 cm^2^ were excised from each carcass. These samples were then placed in an insulated container with an ice pack and transported to the laboratory in refrigerating storage conditions (0–4 °C) within 24 h.

### 2.2. Salmonella spp. Isolation

The isolation procedure followed the guidelines outlined in the ISO 6579:2002 [34] standard. Briefly, using a laboratory blender (Stomacher 400, Seward Ltd., Worthing, England, UK), 25 g of the sample was homogenized in 225 mL of buffered peptone water for about two minutes. The sample was then incubated at 37 °C for 18 hours to facilitate pre-enrichment. After that, 10 mL of Rappaport-Vassiliadis broth (Hi Media) was mixed with 0.1 mL of the pre-enriched inoculum for selective enrichment, and the mixture was incubated for 24 h at 42 °C. After enrichment, the inoculum was spread onto xylose lysine deoxycholate (XLD) agar (Hi Media) by the loopful (10 µL) and incubated for 24 h at 37 °C. On XLD agar, specific *Salmonella* colonies (4–5 colonies/plate) were identified by a somewhat transparent red halo with a black center around a pink-red zone. These colonies were then subjected to additional biochemical characterization. As directed by the manufacturer, biochemical testing using a VITEK 2 GP Immunodiagnostic Assay System (Biomerieux/France) was also used to confirm the presence of Salmonella.

### 2.3. Serotyping

Commercially available antisera from the *Salmonella* antisera test group (Denka Seiken Co., Ltd., Tokyo, Japan) were used for serotyping because they include particular agglutinins for each *Salmonella* antigen. The testing was performed following the method indicated by the manufacturer.

### 2.4. The Extraction of DNA from Colonies

With very minor adjustments, the manufacturer’s technique for employing InstaGene Matrix (BIO-RAD, 732-6030) to extract bacterial DNA was followed. In summary, after resuspending two colonies in 150 µL of 6% (*w/v*) Chelex resin solution (Merck, Darmstadt, Germany), the tubes were incubated for 20 min at 56 °C and 1500 vibrations per minute using a thermomixer. Finally, the tubes were placed at 98 °C for 15 min. After five minutes of centrifuging the samples at 12,000× *g* rpm, the supernatant was kept at −18 °C until it was needed.

### 2.5. Salmonella spp. Differentiation and Confirmation Using Polymerase Chain Reaction (PCR)

PCR targeting the *hilA* (F: 5′-CGGAACGTTATTTGCGCCATGCTGAGGTAG-3′, R: 5′-GCATGGATCCCCGCCGGCGAGATTGTG-3′) and *ompC* (F: 5′-ATCGCTGACTTATGCAATCG-3′, R: 5′-CGGGTTGCGTTATAGGTCTG-3′) genes of Salmonella, as previously reported by Modaressi and Thong [35], was utilized to confirm the identity of the presumptive Salmonella. The amplification was carried out in a final volume of 25 μL, containing 12.5 μL of MasterMix (Bioline, London, UK), 4 μL of DNA template, 6.5 μL of PCR-grade water (Sigma, Saint Louis, MO, USA), and 25 pmol of each primer. There was a negative control for every experiment that used identical reagents except for the DNA template. The positive control used was *Salmonella* Typhimurium ATCC 14028, provided by the regional Sanitary Veterinary Laboratory of Cluj County, Romania. The Taq polymerase and certain primers were used to optimize the PCR conditions. The technique included a 4 min initial denaturation stage at 95 °C, 35 cycles of denaturation (30 s at 95 °C), annealing (30 s at 58 °C), and extension (1 min at 72 °C), and a 5 min final elongation step at 72 °C. Each experiment included a negative control with the same reaction mixture but without the DNA template.

To differentiate between *Salmonella* Typhimurium and *Salmonella* Enteritidis, a multiplex PCR assay was employed, utilizing primers specific for *S*. Enteritidis (ENTF: 5′-TGTGTTTTATCTGATGCAAGAGG-3′, ENTR: 5′-TGAACTACGTTCGTTCTTCTGG-3′, 304 bp) and *S*. Typhimurium (STMF: 5′-TTGTTCACTTTTTACCCCTGAA-3′, STMR: 5′-CCCTGACAGCCGTTAGATATT-3′, 401 bp), as previously described by Modaressi and Thong (2010) [35].

A final volume of 25 μL was used for PCR reactions, which included an optimal mixture of 1 μL of each primer, 12.5 μL of MasterMix (Bioline, London, England, UK), 4 μL of DNA template, and 2.5 μL of PCR-grade water. A preliminary denaturation stage of 94 °C for 4 min was part of the PCR process. This was followed by 30 cycles of denaturation at 94 °C for 30 s, annealing at 58 °C for 30 s, and extension at 72 °C for 1 min. The final elongation step was conducted at 72 °C for 4 min. Strains of *S.* Enteritidis ATCC 13,076 and *S.* Typhimurium ATCC 14,028 were utilized as positive controls, provided by the Sanitary Veterinary Laboratory of Cluj County, Romania. 

### 2.6. Susceptibility Testing

The isolated *Salmonella* strains were subjected to susceptibility testing using the standard disk diffusion technique with the Clinical and Laboratory Standards Institute’s (CLSI) recommended practices [36]. Eleven antimicrobials, some of which are often used in both human and animal medicine, were tested against the isolates. Ampicillin (AMP, 10 μg), cefotaxime (CTX, 30 μg), ceftazidime (CAZ, 30 μg), ciprofloxacin (CHL, 30 μg), gentamicin (GEN, 10 μg), nalidixic acid (NA, 30 μg), streptomycin (S, 10 μg), sulfamethoxazole (SMX, 300 μg), trimethoprim/sulfamethoxazole (SXT, 1.25/23.75 μg), and tetracycline (TET, 30 μg) were among the antimicrobials tested. CLSI breakpoints served as the basis for the results’ interpretation.

### 2.7. Statistical Analysis

The chi-squared test statistical analysis was carried out using OriginPro 8.5 software (OriginLab Corporation, Northampton, MA, USA). A significance value of *p* < 0.05 was used for the results’ interpretation.

## 3. Results

### 3.1. Prevalence and Serotype of Salmonella spp. Isolated in the Traditionally Obtained Pork Meat

Out of the 208 samples examined in this investigation, 48 isolates of *Salmonella* spp., or 23.07%, were discovered. The eight serovars of *Salmonella enterica* subsp. *enterica* that were serotyped from all of the isolates were *S.* Infantis (*n* = 15), *S*. Typhimurium (*n* = 15), *S.* Derby (*n* = 11), *S*. Virkow (*n* = 2), *S*. Brandenburg (*n* = 2), *S*. Ruzizi (*n* = 1), *S*. Muenchen (*n* = 1), and *S*. Bredeney (*n* = 1). The isolates were all members of the subspecies enterica. The serotyping results can be seen in Table 1.

### 3.2. Molecular Confirmation of Salmonella Serotype

PCR confirmation of the Salmonella isolates yielded the expected amplicon sizes of 784 bp for the *hilA* gene (Figure S1, Supplementary File) and 204 bp for the *ompC* gene (Figure S1, Supplementary File). These findings confirmed the presence of both the *hilA* and *ompC* genes in all tested Salmonella isolates. Among the 48 Salmonella isolates, 31.25% (*n* = 15) tested positive for the Typhimurium-specific sequence (401 bp), while none of them were positive for the Enteritidis-specific sequence (304 bp) (Figure S2, Supplementary File). There was consistency between the PCR assay results and traditional serotyping in identifying *S. Typhimurium*.

### 3.3. Antimicrobial Resistance Profiles of Salmonella Isolates

The antimicrobial resistance test results for 48 *Salmonella* isolates are presented in Table 2. Isolates that demonstrated resistance to three or more antimicrobials were categorized as multidrug-resistant (MDR). All *Salmonella* isolates showed resistance to at least one antimicrobial agent, with 45 (93.75%) being classified as multidrug-resistant (MDR). The most commonly observed resistances were to streptomycin (91.66%), tetracycline (83.33%), sulfamethoxazole (68.75%), and nalidixic acid (56.25%). Markedly lower resistance rates were observed for chloramphenicol (18.75%) and ceftazidime (6.25%). None of the samples showed resistance to gentamicin or cefotaxime. Among isolates resistant to various antimicrobial drugs, the serovars Infantis, Derby, and Typhimurium were the most common ones. Fourteen of the fifteen *S*. Typhimurium isolates were multidrug-resistant (MDR), meaning they were resistant to more than three antibiotics (Table 3). It was found that there were 24 distinct resistance patterns among the 46 MDR *Salmonella* isolates, the majority of which were represented by two strains. The majority of the isolates—more than 60%—resisted five or more antibiotics. Two isolates (one isolate of *S.* Brandenburg and an *S.* Virkow isolate) were resistant to seven antimicrobials, and one strain of *S.* Bredeney showed resistance to eight antimicrobials, the greatest number of resistance characteristics among all isolates. Table 3 lists the patterns of antimicrobial resistance that isolates of *Salmonella* displayed.

## 4. Discussion

Between 2017 and 2020, the percentage of pork carcasses in the European Union that tested positive for *Salmonella* varied between 3.1% and 3.9% [37]. Our study has revealed a prevalence of *Salmonella* spp. isolates in pig carcasses of 23.07%, which, compared to other studies conducted in European countries, is significantly higher. For instance, Spain reported the highest frequency of Salmonella-positive pork carcasses in 2020 at 14.3% [38].

Compared to other studies from certain non-European countries, our results showed similar prevalences of *Salmonella*. For instance, Yokozawa et al. (2016) [39] reported a 25.0% prevalence of *Salmonella* in pig carcasses in Vietnam, and Jiu et al. (2020) [40] recorded a 22.9% prevalence in pigs, including carcasses, at commercial slaughterhouses in China. The higher prevalence observed in our study is likely attributable to the collection of samples from carcasses obtained through traditional slaughtering methods, which pose significant hygiene risks during the dressing and evisceration processes.

The most commonly reported *Salmonella* serotypes in people in the EU were *S*. Enteritidis, *S*. Typhimurium, *S*. Infantis, and *S*. Derby, according to the EFSA & ECDC (2021) [39].

The serotypes Enteritidis and Typhimurium, which are frequently connected to consuming pork-containing meals, have been connected to human salmonellosis outbreaks as well as occasional cases. Among 18 pork-related salmonellosis outbreaks, 61.1% were caused by these strains [40]. Despite *S.* Derby being the fifth most common serotype in human infections and accounting for 21.3% of pork isolates, it caused only 1.2% of human salmonellosis cases. Goldcoast and Muenchen, two more uncommon serotypes, have also been connected to diseases associated with pork [41].

PCR offers a cost-effective and rapid method for confirming the presence of *Salmonella*, as shown also by the protocol applied in this study, complementing traditional culture techniques. In this research, the confirmation of presumptive *Salmonella* relied on the detection of the *hilA* and *ompC* genes, both of which are highly specific to *S.* enterica [35]. The use of specific primers for *S.* Enteritidis and *S.* Typhimurium enabled swift identification of these common serovars. In our study, the PCR method indicated that a significant proportion (31.25%; *n* = 15) tested positive for the *S.* Typhimurium-specific sequence. However, the PCR method did not detect any positive samples for the *S.* Enteritidis-specific sequence, thereby corroborating the results obtained through the classical serotyping protocol. The results have evidenced that the highest prevalences among *Salmonella* serotypes in pork carcasses obtained from traditional slaughtering in Romania are reached by Infantis (31.25%) and Typhimurium (31.25%), which pose great health concerns for human consumption. The *S.* Derby serotype, which is said to be most common in pork specimens, was isolated in 11 (22.91%) samples. Molecular confirmation via PCR provided robust validation of traditional serotyping methods, reinforcing the study’s reliability. The ability to quickly detect *S.* Typhimurium highlights the importance of molecular techniques in outbreak response. However, the absence of *S.* Enteritidis in the isolates, despite its prominence in human infections, raises questions about the ecological niche and transmission dynamics of *Salmonella* in local pork production systems. Further studies are warranted to explore this discrepancy and assess the broader implications for public health.

The primary concern with these bacteria extends beyond the diseases they cause in humans to include their increasingly troubling resistance profiles observed over recent decades. Intensive farming practices and the absence of robust antibiotic therapy surveillance systems in traditional farming have frequently resulted in the extensive use of antimicrobials. This has led to widespread antimicrobial resistance (AMR) among microorganisms isolated from food-producing animals. This resistance can be transmitted to humans through direct contact or consumption of animal-derived food products [42]. In many studies, *Salmonella* isolates from pork were commonly resistant to ampicillin, sulfamethoxazole, and tetracycline, reflecting their long-term use in treating pig infections. In 2018/2019, resistance rates for these antimicrobials in *Salmonella* isolates from pig carcasses were 52.7%, 52.1%, and 48.9%, respectively [37]. According to the European Medicines Agency, these antimicrobials are categorized as Category D (Prudence) and should be used as first-line treatments only when necessary [43].

In this study, many isolates exhibited resistance to tetracycline (83.33%) and streptomycin (91.66%), consistent with findings from previous studies [44]. The high resistance rates to these antibiotics are expected, given their extensive use in the Romanian husbandry industry [27]. Interestingly, more than half of the isolates had multidrug-resistant (MDR) profiles; MDR *Salmonella* infections were more common throughout the process of slaughtering. Improved sanitation control strategies in traditional farming systems and slaughtering are essential to enhance the safety of animal products. The alarming rise in resistance to sulfamethoxazole and trimethoprim, essential antimicrobials for treating bacterial infections in humans and animals, is particularly concerning. Despite the prohibition or restriction of unsupervised antibiotic treatment in livestock in many countries, including Romania, this issue persists, raising questions about the effectiveness of these regulatory systems. This study underscores the critical need for rigorous antimicrobial stewardship in Romanian pig farming, along with stringent hygienic practices to prevent microbial contamination. It highlights the urgent necessity for more effective measures, especially regarding traditional slaughtering practices common in Romania, to mitigate antimicrobial resistance and promote sustainable farming practices.

## 5. Conclusions

The study demonstrates a significant prevalence of *Salmonella* spp. in traditionally obtained pork meat, with 23.07% of 208 samples testing positive. Eight serovars were identified, notably *S*. Infantis and *S.* Typhimurium, each present in 15 isolates. The molecular confirmation using PCR indicated that 31.25% of isolates were positive for *S*. Typhimurium, while none were positive for *S.* Enteritidis, validating the traditional serotyping results. Alarmingly, 93.75% of the isolates exhibited multidrug resistance, primarily to antibiotics such as streptomycin and tetracycline, raising serious public health concerns. The antimicrobial resistance (AMR) profiles of these isolates showed extensive resistance, particularly to tetracycline and streptomycin, with over 93% of isolates classified as multidrug-resistant (MDR). This resistance is likely due to the extensive use of antimicrobials in the Romanian husbandry industry. The study underscores the critical need for improved antimicrobial stewardship and stringent hygienic practices, particularly in traditional slaughtering processes, to mitigate the spread of AMR and ensure the safety of animal products. Enhanced sanitation control strategies in traditional slaughtering systems are essential to address this public health concern and promote sustainable farming practices.

## Figures and Tables

**Table 1:** Serotype of *Salmonella* isolates.

Serotype	No.	%
Infantis	15	31.25
Typhimurium	15	31.25
Derby	11	22.91
Ruzizi	1	2.08
Virkow	2	4.16
Brandenburg	2	4.16
Bredeney	1	2.08
Muenchen	1	2.08
Total	48	100

**Table 2:** Antimicrobial resistance in isolates of *Salmonella* from pork meat.

Antibiotic	Number of Resistant and Intermediate Resistant Strains (*n* = 48)	
Β-lactams		
Ampicillin	18 (37.5)	0
Cefotaxime	0	0
Ceftazidime	7 (14.58)	0
Aminoglycosides		
Gentamycin	0	0
Streptomycin	34 (70.83)	0
Sulfonamides		
Sulfamethoxazole	37 (77.08)	0
Sulfamethoxazole/Trimethoprim	8 (16.66)	0
Quinolones and fluoroquinolones		
Nalidixic acid	12 (25)	0
Ciprofloxacin	4 (8.33)	11 (22.91)
Tetracycline	45 (83.33)	0
Chloramphenicol	12 (25)	7 (14.58)

**Table 3:** Patterns of antimicrobial resistance displayed by isolates of *Salmonella*.

Multiple-Resistant Pattern	Serovar	Resistance Pattern	No. of Isolates (%)
One type of antimicrobial	Typhimurium	AMP	1 (2.08)
Two types of antimicrobials	Infantis	AMP, S	1 (2.08)
	Derby	AMP, S	1 (2.08)
Three types of antimicrobials	Typhimurium, Infantis	SMX, NA, S	3 (6.25)
	Derby	SXT, AMP, TET	1 (2.08)
	Derby, Typhimurium	SMX, S, TET	2 (4.16)
	Ruzizi	S, AMP, TET	1 (2.08)
	Infantis	S, SMX, CAZ	1 (2.08)
	Infantis	SXT, AMP, SMX	1 (2.08)
Four types of antimicrobials	Derby	S, NA, CIP, TET	4 (8.33)
	Infantis, Derby, Virkow, Muenchen	SXT, NA, S, TET	4 (8.33)
	Infantis, Typhimurium	SMX, AMP, S, TET	3 (6.25)
Five types of antimicrobials	Derby, Infantis	S, NA, CIP, SMX, TET	5 (10.41)
	Infantis	SMX, NA, CIP, TET, SXT	2 (4.16)
	Typhimurium	SMX, CHL, S, AMP, TET	2 (4.16)
	Typhimurium	SMX, S, AMP, TET, SXT	4 (8.33)
	Derby	SMX, NA, S, CHL, TET	2 (4.16)
	Derby	SMX, NA, S, SXT, TET	2 (4.16)
	Typhimurium	SMX, NA, S, CHL, TET	1 (2.08)
	Typhimurium	SXT, NA, S, CAZ, TET	2 (4.16)
	Brandenburg	SMX, S, CIP, AMP, TET	1 (2.08)
Six types of antimicrobials	Derby, Infantis	SMX, S, CHL, AMP, TET, SXT	2 (4.16)
	Typhimurium,	SMX, S, CHL, AMP, SXT, TET	2 (4.16)
	Infantis	SMX, NA, S, AMP, SXT, TET	1 (2.08)
Seven types of antimicrobials	Brandenburg	SMX, NA, S, CIP, AMP, SXT, TET	1 (2.08)
	Virkov	SMX, NA, S, CIP, SXT, TET	1 (2.08)
Eight types of antimicrobials	Bredeney	SMX, NA, S, CIP, CHL, SXT, TET	1 (2.08)
Total			48 (100%)

SMX, sulfamethoxazole; NA, nalidixic acid; CIP, ciprofloxacin; S, streptomycin; TET, tetracycline; AMP, ampicillin; SXT, Sulfamethoxazole/Trimethoprim; CHL-chloramphenicol; CAZ, ceftazidime; GEN, gentamicin; CTX, cefotaxime.

## Data Availability

Data are available on request due to restrictions.

## Supplementary Materials

The following supporting information can be downloaded at https://www.mdpi.com/article/10.3390/microorganisms12112196/s1: Figure S1: The electrophoretic profile of the *hilA* gene (784 bp) and the *ompC* gene (204 bp) characteristic for *Salmonella* spp. Confirmation; Figure S2: The electrophoretic profile of the *S.* Typhimurium-specific sequence (401 bp).

## References

[1] Muteeb G., Rehman M.T., Shahwan M., Aatif M. (2023). Origin of Antibiotics and Antibiotic Resistance, and Their Impacts on Drug Development: A Narrative Review. Pharmaceuticals.

[2] Salam M.A., Al-Amin M.Y., Salam M.T., Pawar J.S., Akhter N., Rabaan A.A., Alqumber M.A.A. (2023). Antimicrobial Resistance: A Growing Serious Threat for Global Public Health. Healthcare.

[3] O’Neill J. (2014). Review on Antimicrobial Resistance Antimicrobial Resistance: Tackling a Crisis for the Health and Wealth of Nations. London: Review on Antimicrobial Resistance. https://amr-review.org/sites/default/files/AMR%20Review%20Paper%20-%20Tackling%20a%20crisis%20for%20the%20health%20and%20wealth%20of%20nations_1.pdf.

[4] Koutsoumanis K., Allende A., Álvarez-Ordóñez A., Bolton D., Bover-Cid S., Chemaly M., Davies R., De Cesare A., Herman L., Hilbert F. (2021). Role played by the environment in the emergence and spread of antimicrobial resistance (AMR) through the food chain. EFSA J..

[5] Carrasco E., Morales-Rueda A., García-Gimeno R.M. (2012). Cross-contamination and recontamination by *Salmonella* in foods: A review. Food Res. Int..

[6] Swartz M.N. (2002). Human diseases caused by foodborne pathogens of animal origin. Clin. Infect. Dis..

[7] Vencia W., Gariano G.R., Bianchi D.M., Zuccon F., Sommariva M., Nguon B., Malabaila A., Gallina S., Decastelli L. (2015). A *Salmonella Enterica* Subsp. *Enterica* Serovar Enteritidis Foodborne Outbreak after Consumption of Homemade Lasagne. Ital. J. Food Saf..

[8] Galanis E., Lo Fo Wong D.M., Patrick M.E., Binsztein N., Cieslik A., Chalermchikit T., Aidara-Kane A., Ellis A., Angulo F.J., Wegener H.C. (2006). Web-based surveillance and global *Salmonella* distribution, 2000-2002. Emerg. Infect. Dis..

[9] Yan L., Alam M.J., Shinoda S., Miyoshi S., Shi L. (2010). Prevalence and antimicrobial resistance of *Salmonella* in retail foods in northern China. Int. J. Food Microbiol..

[10] Brands D.A., Inman A.E., Gerba C.P., Mare C.J., Billington S.J., Saif L.A. (2005). Prevalence of *Salmonella* spp. in oysters in the United States. Appl. Environ. Microbiol..

[11] Zhao S., White D.G., Friedman S.L., Glenn A., Blickenstaff K., Ayers S.L., Abbott J.W., Hall-Robinson E., McDermott P.F. (2008). Antimicrobial resistance in *Salmonella enterica* serovar Heidelberg isolates from retail meats, including poultry, from 2002 to 2006. Appl. Environ Microbiol..

[12] Hur J., Jawale C., Lee J.H. (2012). Antimicrobial resistance of *Salmonella* isolated from food animals: A review. Food Res. Int..

[13] Koh Y., Bae Y., Lee Y.S., Kang D.H., Kim S.H. (2022). Prevalence and characteristics of *Salmonella* spp. isolated from raw chicken meat in the Republic of Korea. J. Microbiol. Biotechnol..

[14] Griffith R.W., Carlson S.A., Krull A.C., Zimmerman J.J., Karriker L.A., Ramirez A., Schwartz K.J., Stevenson G.W., Zhang J. (2019). Salmonellosis. Diseases of Swine.

[15] Fedorka-Cray P.J., Gray J.T., Wray C., Wray C., Wray A. (2000). Salmonella Infections in Pigs. Salmonella in Domestic Animals.

[16] Andino A., Hanning I. (2015). *Salmonella enterica*: Survival, colonization, and virulence differences among serovars. Sci. World J..

[17] Carvajal A., Kramer M., Argüello H. (2024). Salmonella Control in Swine: A Thoughtful Discussion of the Pre- and Post-Harvest Control Approaches in Industrialized Countries. Animals.

[18] Sinh D.X., Hung N.V., Phuc P.D., Unger F., Ngan T.T., Grace D., Makita K. (2019). Risk factors associated with *Salmonella* spp. prevalence along smallholder pig value chains in Vietnam. Int. J. Food Microbiol..

[19] Asghari A., Sadrebazzaz A., Shamsi L., Shams M. (2021). Global prevalence, subtypes distribution, zoonotic potential, and associated risk factors of *Blastocystis* sp. in domestic pigs (*Sus domesticus*) and wild boars (*Sus scrofa*): A systematic review and meta-analysis. Microb. Pathog..

[20] Alarcón L.V., Allepuz A., Mateu E. (2021). Biosecurity in pig farms: A review. Porc. Health Manag..

[21] Ojha S., Kostrzynska M. (2007). Approaches for reducing *Salmonella* in pork production. J. Food Prot..

[22] Campos J., Mourão J., Peixe L., Antunes P. (2019). Non-typhoidal *Salmonella* in the pig production chain: A comprehensive analysis of its impact on human health. Pathogens.

[23] Soliani L., Rugna G., Prosperi A., Chiapponi C., Luppi A. (2023). *Salmonella* infection in pigs: Disease, prevalence, and a link between swine and human health. Pathogens.

[24] Deane A., Murphy D., Leonard F.C., Byrne W., Clegg T., Madigan G., Griffin M., Egan J., Prendergast D.M. (2022). Prevalence of *Salmonella* spp. in slaughter pigs and carcasses in Irish abattoirs and their antimicrobial resistance. Ir. Vet. J..

[25] Smid J.H., van Hoek A.H.A.M., Aarts H.J.M., Havelaar A.H., Heres L., de Jonge R., Pielaat A. (2014). Quantifying the sources of *Salmonella* on dressed carcasses of pigs based on serovar distribution. Meat Sci..

[26] Bearson B.L., Trachsel J.M., Holman D.B., Brunelle B.W., Sivasankaran S.K., Simmons M., Wasilenko J., Tillman G., Johnston J.J., Bearson S.M.D. (2019). Complete Genome Sequence of Multidrug-Resistant *Salmonella enterica* Serovar I 4,[5],12:i:—2015 U.S. Pork Outbreak Isolate USDA15WA-1. Microbiol. Resour. Announc..

[27] Mihaiu L., Lapusan A., Tanasuica R., Sobolu R., Mihaiu R., Oniga O., Mihaiu M. (2014). First study of *Salmonella* in meat in Romania. J. Infect. Dev. Ctries..

[28] Castellanos L.R., van der Graaf-van Bloois L., Donado-Godoy P., León M., Clavijo V., Arévalo A., Bernal J.F., Mevius D.J., Wagenaar J.A., Zomer A. (2018). Genomic characterization of extended-spectrum cephalosporin-resistant *Salmonella enterica* in the Colombian poultry chain. Front. Microbiol..

[29] Centers for Disease Control and Prevention (CDC) (2017). *Salmonella* page. https://www.cdc.gov/salmonella/general/technical.html.

[30] Pan H., Paudyal N., Li X., Fang W., Yue M. (2018). Multiple food-animal-borne route in transmission of antibiotic-resistant *Salmonella Newport* to humans. Front. Microbiol..

[31] World Health Organization (WHO) (2017). Model List of Essential Medicines. https://iris.who.int/handle/10665/273826.

[32] World Health Organization (WHO) (2024). WHO Bacterial Priority Pathogens List, 2024. https://iris.who.int/bitstream/handle/10665/376776/9789240093461-eng.pdf?sequence=1.

[33] (2007). Commission Regulation (EC) No 1441/2007 of 5 December 2007 amending Regulation (EC) No 2073/2005 on microbiological criteria for foodstuffs. Off. J. Eur. Union.

[34] Horizontal Method for the Detection of Salmonella, Including Salmonella Typhi and Salmonella Paratyphi.

[35] Modarressi S.H., Thong K.L. (2010). Isolation and molecular subtyping of *Salmonella enterica* from chicken, beef, and street foods in Malaysia. Sci. Res. Essays.

[36] Clinical and Laboratory Standards Institute (2018). Performance Standards for Antimicrobial Susceptibility Testing.

[37] Roasto M., Bonardi S., Mäesaar M., Alban L., Gomes-Neves E., Vieira-Pinto M., Vågsholm I., Elias T., Lindegaard L.L., Blagojevic B. (2023). *Salmonella enterica* prevalence, serotype diversity, antimicrobial resistance, and control in the European pork production chain. Trends Food Sci. Technol..

[38] European Food Safety Authority (EFSA) and European Centre for Disease Prevention and Control (ECDC) (2021). The European Union Summary Report on Antimicrobial Resistance in zoonotic and indicator bacteria from humans, animals, and food in 2018/2019. EFSA J..

[39] Yokozawa T., Sinh D.X., Hung N.V., Lapar L., Makita K. (2016). Transition of *Salmonella* prevalence in pork value chain from pig slaughterhouse to markets in Hung Yen, Vietnam. J. Vet. Epidemiol..

[40] Jiu Y., Meng X., Hong X., Huang Q., Wang C., Chen Z., Zhao L., Liu X., Lu Y., Li S. (2020). Prevalence and characterization of *Salmonella* in three typical commercial pig abattoirs in Wuhan, China. Foodborne Pathog. Dis..

[41] Almansour A.M., Alhadlaq M.A., Alzahrani K.O., Mukhtar L.E., Alharbi A.L., Alajel S.M. (2023). The silent threat: Antimicrobial-resistant pathogens in food-producing animals and their impact on public health. Microorganisms.

[42] European Medicines Agency (EMA) (2020). Categorisation of Antibiotics in the European Union. https://www.ema.europa.eu/en/documents/report/categorisation-antibiotics-european-union-answer-request-european-commission-updating-scientific_en.pdf.

[43] Tang B., Elbediwi M., Nambiar R.B., Yang H., Lin J., Yue M. (2022). Genomic Characterization of Antimicrobial-Resistant *Salmonella enterica* in Duck, Chicken, and Pig Farms and Retail Markets in Eastern China. Microbiol. Spectr..

[44] Chen Z., Biswas S., Aminabadi P., Stackhouse J.W., Jay-Russell M.T., Pandey P.K. (2019). Prevalence of *Escherichia coli* O_157_ and *Salmonella* spp. in Solid Bovine Manure in California Using Real-Time Quantitative PCR. Lett. Appl. Microbiol..

