# Supplementary material for: Evaluation of Multidrug Resistance of Salmonella Isolated from Pork Meat Obtained from Traditional Slaughter Systems in Romania
Source: Microorganisms. 2024 Oct 30;12(11):2196. doi: 10.3390/microorganisms12112196 (PMC11596094; doi:10.3390/microorganisms12112196)
Supplement: Supplementary file 1 [file microorganisms-12-02196-s001.zip › microorganisms-3238110-supplementary.pdf]

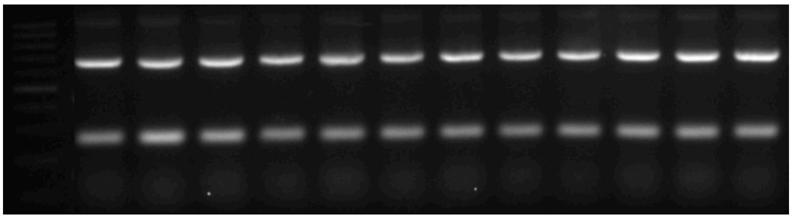

Figure S1: The electrophoretic profile of the hilA gene (784 bp) and the ompC gene (204 bp) characteristic for *Salmonella* spp. confirmation

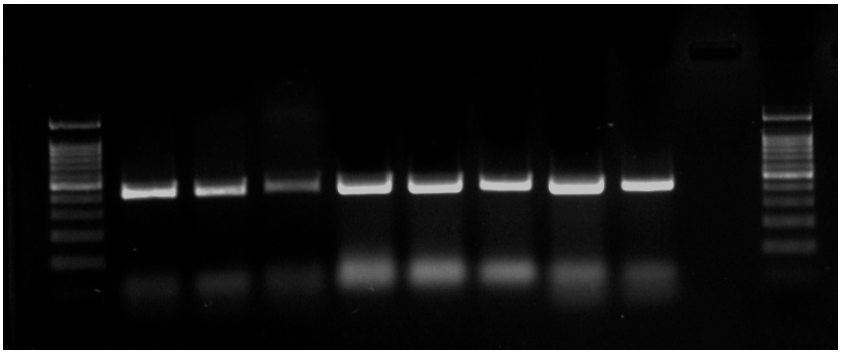

Figure S2: The electrophoretic profile of the *S.* Typhimurium-specific sequence (401 bp)
